# From Stigma to Therapy: Pharmacological Insights into Saffron Bioactives for Major Non-Communicable Diseases

**DOI:** 10.3390/ph19030484

**Published:** 2026-03-15

**Authors:** Catarina Campos, Yahya Ramadan Elfardi, El Mehdi Darrag, Hassan Laouane, Rosa Perestrelo, Latifa Bouissane, José S. Câmara

**Affiliations:** 1 CQM-Centro de Química da Madeira, Universidade da Madeira, Campus da Penteada, 9020-105 Funchal, Portugal; catarina.campos@staff.uma.pt (C.C.); rmp@staff.uma.pt (R.P.); 2 Sustainable Processes, Advanced Materials and Computational Chemistry Team, Polydisciplinary Faculty of Beni Mellal, Sultan Moulay Slimane University, P.O. Box 592 Mghila, Beni-Mellal 23000, Morocco; yahyaramadan.elfardi@usms.ma; 3 Laboratory of Biomolecular and Medicinal Chemistry, Department of Chemistry, Faculty of Sciences Semlalia, Marrakesh 40001, Morocco; elmehdi.darrag@gmail.com (E.M.D.); h.laouane@uca.ac.ma (H.L.); 4 Departamento de Química, Faculdade de Ciências Exatas e da Engenharia, Universidade da Madeira, Campus Universitário da Penteada, 9020-105 Funchal, Portugal

**Keywords:** *Crocus sativus* L., saffron, bioactive compounds, therapeutic agents, pharmacological applications, non-communicable diseases

## Abstract

*Crocus sativus* L. (saffron), a sterile geophyte of the Iridaceae family, has been traditionally used in culinary and medicinal practices and is currently gaining attention as a source of pharmacologically active metabolites. The main bioactive compounds (BACs) of saffron, crocin, crocetin, picrocrocin, and safranal, are associated with a wide range of biological activities, including anti-inflammatory, antioxidant, immunomodulatory, antimicrobial, antiproliferative, and antidiabetic properties, among others. This review aims to comprehensively and critically summarize the preclinical and clinical evidence for saffron-derived BACs in the context of the most prevalent non-communicable diseases. A literature search of the main scientific databases was conducted to identify peer-reviewed articles on neurodegenerative disorders, cancer, cardiovascular diseases, and diabetes mellitus, with additional topics on ethnopharmacology, phytochemistry, safety, and toxicity. The mechanistic findings include anti-inflammatory, immunomodulatory, antioxidant, antiproliferative, and neuroprotective effects, mediated by activation of the Nrf2 pathway and inhibition of NF-κB. Eligibility criteria were applied, excluding publications focused primarily on food, cosmetics, or technological applications, to prioritize mechanistic and therapeutic endpoints. The findings suggest that BACs from saffron extracts have promising disease-modifying properties and symptom-relieving actions, especially in the case of neurologic disorders, mild cognitive impairment, and some models of metabolic and oncological diseases. Nevertheless, the current variability in study design, dosage, standardization of plant extracts, and sample size limits a conclusive clinical application. More carefully designed studies with a representative number of cases and well-defined plant preparations are needed to validate efficacy, establish structure-activity relationships, and define the prevention and therapeutic potential of saffron in evidence-based pharmacotherapy.

## 1. Introduction

Natural products remain one of the main sources of pharmacologically bioactive compounds (BACs) and have played a fundamental role in the development of new drugs. Plant-derived metabolites have been extensively investigated due to their structural diversity, biological activity, and relatively positive safety properties. *Crocus sativus* L. of the Iridaceae family, commonly known as saffron, has garnered substantial attention among medicinal plants due to the presence of a diversity of BACs and its established therapeutic properties [1]. Saffron is one of the most expensive spices in the world, due to intense and time-consuming manual cultivation and the number of flowers required for production, factors that contribute to its limited availability and pharmaceutical relevance [2,3,4,5,6].

Many civilizations have long employed saffron to alleviate a variety of illnesses, such as gastrointestinal issues and inflammatory conditions, among others. In recent years, a growing body of scientific evidence supporting several of these traditional uses has shown the potential of saffron and its main ingredients—crocins, crocetin, safranal, and picrocrocin—as pharmacologically relevant compounds. Numerous in vitro and in vivo studies have demonstrated that these BACs possess antioxidant, anti-inflammatory, neuroprotective, anticancer, and cardioprotective properties [2,7,8,9,10,11].

The biological activities of saffron are specifically described by its ability to modulate the important molecular mechanisms involving oxidative stress, inflammation, apoptosis, and neurotransmission. Several studies have shown that the BACs of saffron can modulate the neurotransmitter systems involving serotonin and dopamine, as well as the signaling mechanisms. The rising interest in saffron as a potential candidate for the development of novel therapeutic strategies, particularly in the treatment of neurological disorders, cancer, and chronic inflammatory diseases, has a solid basis in these mechanisms [10,12,13,14]. The translation of saffron BACs to pharmaceutical uses remains a challenge, despite the increasing number of preclinical and clinical trials. The use of BACs in clinical settings is currently challenged by concerns regarding absorption, pharmacokinetics, uniformity of dosage, and safety for long-term use. Furthermore, the interpretation of results is complicated by the diversity of study designs and the convergence of pharmaceutical and nutraceutical approaches [6,15,16,17,18,19].

The present review provides a comprehensive narrative synthesis of the pharmacological effects of saffron and its principal BACs, with a specific focus on molecular mechanisms, therapeutic relevance, and translational challenges. By integrating evidence from in vitro studies, animal models, and clinical trials, this review critically assesses saffron’s potential as a multi-target pharmacological agent and identifies key limitations and future directions for its development within pharmaceutical and integrative medicine frameworks. Considering the scope of disease areas covered, this review is intended to be a broad narrative overview rather than an exhaustive systematic evaluation of each disease, with a focus on critically evaluating study quality, level of evidence, gaps between preclinical findings and clinical applicability, and limitations across sections.

## 2. Method

This review trails a comprehensive narrative synthesis approach based on a structured and comprehensive literature search conducted in 2025. The keywords “*Crocus sativus*”, “saffron”, “crocin”, “crocetin”, “safranal”, “picrocrocin”, “neurodegenerative diseases”, “mental disorders”, “cancer”, “cardiovascular disease”, “metabolic disorders”, and “clinical trials” were combined and searched on scientific databases such as PubMed, Scopus, Web of Science, and Google Scholar to achieve peer-reviewed articles published between January 2000 and January 2026.

Eligibility criteria were established based on the PICO(S) elements: population (animal models, human participants, and disease-relevant cellular systems); intervention (whole saffron, standardized extracts, parts of saffron, or specific saffron-derived compounds); study type (in vitro, in vivo, and controlled or observational human clinical studies); and outcomes (disease-relevant pharmacological endpoints, namely, biochemical, functional, clinical, and mechanistic). Studies that focused on culinary or general nutraceuticals without disease-focused pharmacodynamics were excluded on the basis of pharmacological relevance. Non-English articles and non-peer-reviewed publications were also excluded. The initial search identified 312 records. After removal of duplicates (*n* = 58), 254 articles were screened by title, abstract, and date of publication. Subsequently, 95 studies were included in the final synthesis.

No formal meta-analysis was performed due to heterogeneity in the study design, formulation, and outcome measurements. Although no standardized risk of bias tool was applied, included studies were critically appraised based on study rigor, extract standardization, endpoint robustness, and translational relevance, with more weight given to controlled clinical trials compared to preclinical studies.

## 3. BACs with Pharmaceutical Relevance

The pharmaceutical potential of saffron is primarily attributed to a restricted number of BACs that have demonstrated consistent activity in both preclinical and clinical studies. Perhaps due to its complex chemical composition, pharmacological research has focused on a small set of BACs, namely crocins, crocetin, safranal, and picrocrocin, which are considered the main drivers of its therapeutic effects (Table 1).

The carotenoid derivatives crocin and its aglycone crocetin, which give saffron its distinctive color, are the most well-investigated BACs from a pharmacological perspective. Crocetin is a lipophilic dicarboxylic acid that results from intestinal hydrolysis, while crocins are water-soluble glycosylated apocarotenoids. According to pharmacological investigations, these BACs demonstrate anti-inflammatory and antioxidant properties, mostly by modulation of inflammatory mediators and oxidative stress pathways. Crocins and crocetin have also been shown to exert neuroprotective, cardioprotective, and anticancer properties by influencing signaling pathways, such as nuclear factor κB (NF-κB), nuclear factor erythroid 2-related factor 2 (Nrf2), and phosphatidylinositol 3-kinase/protein kinase B (PI3K/Akt). Remarkably, crocetin has shown to be more bioavailable than crocins, indicating its potential for drug development [10,12,13,18,19,20]. Another pharmacologically noteworthy BAC that gives saffron its distinctive scent is safranal, a monoterpene aldehyde resulting from saffron processing. Neuroprotective, anticancer, anticonvulsant, antidepressant, and antioxidant properties have all been correlated with safranal. These effects are thought to be mediated by interactions with the central nervous system, involving the regulation of oxidative stress and apoptotic signaling, as well as the modulation of gamma-aminobutyric acid neurotransmission-related (GABAergic) and serotonergic pathways. Safranal is more quickly absorbed due to its volatility and lipophilicity, even though additional research is needed to understand its pharmacokinetic behavior and long-term safety [19,23,24,25].

In contrast, picrocrocin, a glycosylated monoterpene, has received less research attention and is mostly acknowledged for its role in the bitter flavor of saffron. Nonetheless, novel research suggests that picrocrocin may support saffron’s overall pharmacological activity and contribute to its anti-inflammatory and antioxidant characteristics. Rather than acting as a stand-alone medicinal agent, its contribution seems to be especially significant in synergistic combinations with other saffron BACs [19,26,27].

From a structure-activity relationship, the pharmacological effects of saffron BACs are correlated with their chemical structure. The large conjugated polyene backbone of crocetin is responsible for its high redox activity, whereas glycosylation in crocin improves water solubility but reduces passive diffusion across cell membranes. Conversely, the lipophilic and volatile aldehyde group of safranal promotes blood–brain barrier penetration and interaction with neuronal pathways. These structural features interactively modulate affinity for targets, intracellular distribution, metabolic half-life, and ultimately pharmacological response, thus emphasizing the impact of chemical structure in determining therapeutic application [7,9,19].

**Figure 1:**
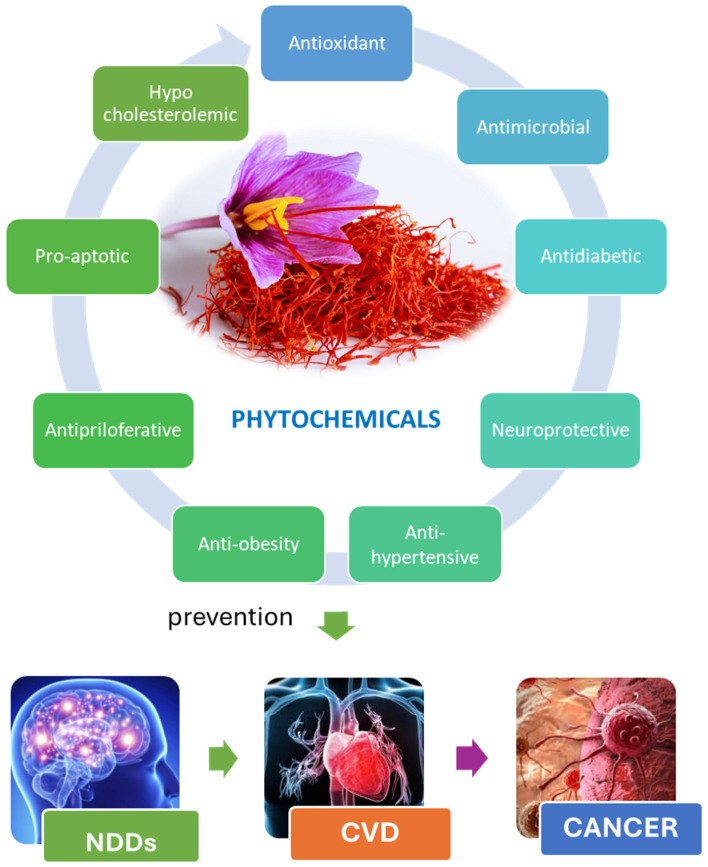
Integrated biological effects of saffron in non-communicable diseases (NDDs, neurodegenerative diseases; CVD, cardiovascular diseases).

Further preclinical research supports the potential meaning of saffron BACs in neurodegenerative and neuropsychiatric illnesses by showing that they have neuroprotective effects through reduction of neuroinflammation, prevention of amyloid aggregation, and modulation of neurotransmitter systems. Inhibition of tumor cell proliferation, activation of apoptosis, and suppression of tumor development and spreading pathways have all been linked to anticancer activity. Furthermore, saffron-derived BACs have demonstrated metabolic and cardioprotective advantages, such as improvement of lipid profiles, reduction of oxidative damage, enhancement of insulin sensitivity, and regulation of blood glucose levels (Figure 1) [14,28,29].

Moreover, saffron contains minor compounds, such as flavonoids (kaempferol, luteolin, and quercetin glycosides), phenolic acids (chlorgenic acid and gallic acid), anthocyanins (delphinidin, cyanidin, and petunidin derivatives), vitamins, minerals (Mg, P, Ca, K, and Na), carbohydrates, and amino acids in addition to these primary BACs [19,30,31]. Phenolic compounds, especially flavonoids, mostly found in saffron tepals, are well known for their antioxidant and anti-inflammatory activities. However, their direct contribution to saffron’s therapeutic properties is restricted by their very low concentrations and the lack of robust pharmacological evidence. Hence, rather than being the main focus of pharmaceutical development, these BACs are regarded as secondary contributors [9,19,27,32].

## 4. Pharmacological Effects of Saffron in Non-Communicable Diseases

Saffron’s main BACs, namely crocin, crocetin, picocrocin, and safranal, possess several pharmacological effects. Several studies have reported that these BACs demonstrated antioxidant, anti-inflammatory, anticancer, antidepressant, hypoglycemic, hypolipidemic, memory-enhancing effects, and many others [3,19].

### 4.1. Neurodegenerative and Neuropsychiatric Disorders

Alzheimer’s disease, Parkinson’s disease, multiple sclerosis, cerebral ischemia, and mental illness are among the distinct groups of conditions recognized as neurodegenerative diseases and related neuropsychiatric disorders. These conditions share common pathological mechanisms, including oxidative stress, chronic neuroinflammation, mitochondrial dysfunction, protein aggregation, excitotoxicity, impaired autophagy, and apoptosis. Nonetheless, the majority of existing treatments are still symptomatic and frequently have negative side effects. Table 2 summarizes the neuroprotective and neuromodulatory properties of saffron and its main BACs.

#### 4.1.1. Alzheimer’s Disease

Alzheimer’s disease is depicted by the deposition of amyloid-beta (Aβ) peptides in neurons, causing the formation of senile plaques, while hyperphosphorylated tau protein accumulation forms neurofibrillary tangles. These pathological processes are enhanced by the increased oxidative stress and activation of apoptosis, including caspase-3-mediated neuronal cell death. Saffron has been proposed to interfere with this particular pathway by blocking the deposition of Aβ plaques and reducing Aβ-induced neurotoxicity [50]. By reducing Aβ-induced synaptic loss, neuronal apoptosis, and dendritic degeneration in vulnerable brain regions such as the hippocampus and frontal cortex, crocin (30 mg/kg/day) has displayed neuroprotective effects in experimental Alzheimer’s disease models, indicating its ability to maintain neuronal architecture and viability [35]. By altering peptide aggregation pathways, BACs derived from saffron directly affect Aβ pathogenesis at the molecular level. In this sense, trans-crocin-4 and crocetin redirected Aβ_1–40_ assembly toward non-toxic species, thereby inhibiting fibrillogenesis and reducing the burden of pathogenic oligomers [33].

Beyond aggregation control, crocetin has emerged as a potent enhancer of Aβ clearance through activation of macroautophagy via the STK11/LKB1 (serine/threonine kinase 11)-mediated AMP-activated protein kinase (AMPK) pathway. In transgenic animal models for Alzheimer’s disease, crocetin has been demonstrated to successfully cross the blood–brain barrier, cause lysosomal degradation of Aβ, alleviate neuroinflammation, and obviously enhance cognitive functions [22]. In mild to moderate patients with Alzheimer’s disease who were undergoing conventional cholinesterase inhibitor treatment, administration of saffron supplements (15 mg, 2 times daily) alleviated systemic levels of oxidative stress and pro-inflammation, implying an additive biochemical effect, even when cognitive functions were not obviously observed [20]. Furthermore, the concomitant pre-administration of a standardized saffron dosage (Repron^®^, 10 mg/kg/day) showed alleviation of the cognitive and visual disturbances caused by neuroinflammation in vivo, which emphasizes the use of anti-inflammatory modulation and the retina as a biomarker in the therapeutic response of Alzheimer’s disease [8].

#### 4.1.2. Parkinson’s Disease

Parkinson’s disease is caused by damage to dopaminergic neurons in the substantia nigra, likely due to oxidative stress, inflammation, and acetylcholinesterase activity, and is associated with tremors, muscle rigidity, imbalance, and delayed movements. It has been reported that lead, a neurotoxic metal element, could contribute to disease development.

In models of toxin and metal-induced Parkinson’s disease, crocin and saffron extracts preserved the integrity of both dopaminergic and noradrenergic systems, restored tyrosine hydroxylase expression in nigrostriatal and mesocorticolimbic pathways, and improved locomotor function significantly, thereby affirming a potent protective function for crocin and saffron extracts against environment-related dopaminergic neurotoxicity in vivo [36]. At the mechanistic level, crocin activates pro-survival signaling cascades, including PI3K/Akt/mTOR, suppresses apoptosis, attenuates α-synuclein accumulation, and enhances dopaminergic neurotransmission. These effects were further reinforced by the upregulation of Parkinson’s disease-relevant microRNAs, including miRNA-7 and miR-NA-221 [38]. In rotenone-induced PD models, crocin alleviated motor deficits, preserved striatal dopamine levels, and mitigated oxidative and inflammatory stress [38]. Translational relevance is supported by current randomized clinical evidence demonstrating that oral crocin supplementation (60 mg/day) improved motor function and activities of daily living in patients with idiopathic Parkinson’s disease when used as an adjunct to standard dopaminergic therapy, with a favorable safety profile [37].

#### 4.1.3. Multiple Sclerosis

Multiple sclerosis is a chronic autoimmune disease characterized by central nervous system demyelination, leading to myelin sheath destruction and impairing neuron signal transmission. It affects physical, mental, cognitive, and motor activity [39].

In a cuprizone-induced model of multiple sclerosis, crocin administration attenuated demyelination-associated motor deficits and depressive-like behaviors, improving locomotor coordination and reflexive motor performance, while normalizing systemic and central oxidative stress markers, including malondialdehyde, superoxide dismutase, glutathione peroxidase, and total antioxidant status. These findings highlight its capacity to counteract oxidative stress-driven oligodendrocyte dysfunction and secondary neuroinflammation [39].

Clinical assays demonstrated that a nanoformulated crocin-selenium complex (Cor@SeNs) improved cognitive performance in multiple sclerosis patients, particularly in verbal learning, memory retention, and information processing speed, alongside enhancements in total antioxidant capacity [40]. The observed clinical efficacy was mechanistically attributed to the synergistic antioxidant actions of crocin and selenium-dependent selenoproteins, combined with improved bioavailability and blood–brain barrier penetration conferred by nanoparticle delivery, enabling effective modulation of redox homeostasis and mitochondrial dysfunction, key upstream drivers of neurodegeneration in multiple sclerosis [40]. Additional clinical evidence suggested that saffron may also exert immunomodulatory effects, as treatment in relapsing-remitting multiple sclerosis patients was associated with reduced circulating matrix metalloproteinase-9 (MMP-9), a key mediator of T-cell migration across the blood–brain barrier, and increased levels of its endogenous inhibitor, tissue inhibitor of metalloproteinases-1 (TIMP-1), indicating attenuation of pathological immune cell trafficking to the central nervous system [41].

#### 4.1.4. Cerebral Ischemia

Cerebral ischemia results from a disturbed blood flow to the brain, leading to neuronal damage through inflammation, oxidative stress, and apoptosis processes. Angiogenesis and neurogenesis are linked with stroke recovery.

In experimental models of focal cerebral ischemia/reperfusion, the administration of saffron improved neurological and motor outcomes, preserved neuronal integrity in cortical and hippocampal regions, and reduced infarct-associated histopathological damage. These effects were associated with reduced lipid peroxidation, nitric oxide production, and caspase-3 and Bax expression, alongside the restoration of endogenous antioxidant defenses [42]. Upregulation of vascular endothelial growth factor (VEGF) suggested enhanced angiogenesis and neurotrophic support as critical contributors to post-ischemic recovery [42].

In a post-stroke model, saffron’s chronic suppressive effects encompassed a reduction in astrogliosis and glial scar tissue within the penumbra, decreased pro-inflammatory cytokine expression accompanied by an increase in anti-inflammatory factors, and promoted neuronal survival and functional recovery, indicating modulation of glial reactions post-stroke [43]. The translational relevance was supported by the existence of clinical trial data that have found that oral aqueous extracts of saffron were correlated with reduced stroke severity and decreased circulating biomarkers of neuronal and glial injury in hospitalized patients, while increasing blood levels of brain-derived neurotrophic factor [44].

#### 4.1.5. Mental Illness

Saffron has been cited in Canadian clinical guidance documents as a potential adjunctive option for mood disorders, based on emerging clinical evidence [51]. Several clinical trials have reported its antidepressant and anxiolytic properties for most age groups, as its principal BAC, crocin, acts as a pleiotropic modulator of mental illness, particularly depressive and anxiety-related disorders.

At the molecular level, crocin exerted antidepressant-like effects through activation of neuroplasticity-associated signaling cascades, notably the BDNF–mTOR–ERK pathway, resulting in enhanced synaptic protein expression, dendritic growth, and reversal of stress-induced depressive behaviors. Pharmacological blockade of mTOR signaling abolishes these effects, confirming pathway dependency [45]. Ex vivo studies in humans suggested the capability of the circulating metabolites produced post-saffron consumption to protect human neurons against the actions of oxidative stress by enhancing the production of brain-derived neurotrophic factor (BDNF), promoting the secretion of dopamine and serotonin, inhibiting the expression of the serotonin transporter, and decreasing serotonin metabolism, which collectively resemble the mechanisms of action of selective serotonin reuptake inhibitor (SSRI) antidepressants [46].

Preclinical behavioral studies demonstrated the capacity of saffron extracts to reduce anhedonia and depression-like responses generated in stress animal paradigms, which depict the involvement of antioxidant and monoaminergic mechanisms in affect regulation [47]. Further clinical trials reported improvements in self-perceived mental health status and quality of life in people experiencing subclinical levels of depression, as measured by changes in amino acid pathways, without evidence of the impact of saffron supplements regarding systemic indices of inflammation as well as hypothalamic-pituitary-adrenal system activity [48]. In patients with major depressive disorder, saffron exhibited antidepressant efficacy comparable to the SSRI sertraline, including older populations, highlighting its clinical relevance and favorable tolerability profile [49].

Despite the research discussed above suggesting potential pharmacological effects of saffron and its BACs, there are certain limitations to these studies. Firstly, most in vitro studies have applied concentrations that may not be within the range of physiological plasma concentrations, following oral administration. Consequently, there are concerns regarding the pharmacological relevance of these studies. Secondly, in vivo studies, while providing positive effects of saffron, have not characterized the pharmacokinetic profile of the BACs. In addition, the formulation of the extract was not standardized in most of these studies. It is also important to note that while conducting in vivo studies, the effects of BACs have not been studied in the scope of human disease. In most of the clinical studies, small sample sizes, short durations of administration, and heterogeneous formulations were assessed. Moreover, the positive effects of BAC in vitro studies suggest the possibility of publication bias. These factors highlight translational gaps and underscore the need for standardized formulations, rigorous pharmacokinetic evaluation, and appropriately powered randomized controlled trials.

### 4.2. Oncological Disease

Saffron has been extensively investigated for its anticancer potential, mainly attributed to its BACs, which act through multiple molecular mechanisms involved in tumor initiation and progression. These mechanisms include the inhibition of DNA and RNA synthesis, suppression of cancer cell proliferation, induction of apoptosis and autophagy, inhibition of angiogenesis and metastasis, and modulation of oncogenes and tumor suppressor gene expression (Table 3).

Saffron and its BACs, especially crocin and safranal, exhibited broad-spectrum anticancer actions across numerous tumor types via pleiotropic and convergent molecular routes. Mechanistic, preclinical, and translational evidence suggests that crocin inhibits key oncogenic signaling pathways, most remarkably the PI3K/Akt/mTOR, thereby inhibiting tumor cell proliferation and inducing apoptosis in hormone-related and solid malignancies. In thyroid and cervical cancer models, reactivation of this pathway reversed the observed antitumor efficacy, proving mechanistic specificity [12,58]. Outside growth inhibition, crocin decreased pro-inflammatory cytokine production in breast cancer cells and downregulates protein kinase C theta (PRKCQ)-dependent nuclear factor kappa B (NF-κB) activation. This disrupts the inflammatory microenvironment that advances tumor growth [13].

Saffron’s anticancer profile is beyond improved by epigenetic and genomic stability mechanisms. In prostate cancer models, saffron treatment induced apoptotic cell death while simultaneously downregulating DNA methyltransferases, DNA repair intermediates, and oncogenic regulators, such as AR, c-Myc, and NF-κB, indicating its capability to target tumor plasticity and resistance pathways [29]. In colorectal cancer models, altering the IL-17/Th17 axis, downregulating PD-L1 expression, and reestablishing the balance of CD4/CD8 T cells within the tumor microenvironment were reported, indicating potential enhancement of immune checkpoint-based immunotherapy [66].

Formulation techniques, as a whole, also influenced therapeutic outcomes. For example, nanoencapsulated crocin increased the sensitivity of triple-negative breast cancer cells to doxorubicin by inducing mitochondrial apoptosis and cell-cycle arrest, which overcomes innate chemoresistance [54]. Safranal was reported to exert antimetastatic effects in aggressive breast cancer, causing apoptosis, mitochondrial dysfunction, and extensive dysregulation of glycoprotein sialylation implicated in cell adhesion, migration, and survival, according to complementary data from proteomic and sialiomic investigations [55]. Crocin’s anti-angiogenic and anti-metastatic properties were further supported by in vivo studies, especially through the suppression of VEGF and MMP-9 signaling. In breast and hepatocellular carcinoma models, respectively, synergistic tumor growth inhibition was observed when mixed with metformin or the multi-kinase inhibitor sorafenib [57,61].

As summarized in Table 3, saffron extracts and their BACs demonstrated antiproliferative, pro-apoptotic, and anti-angiogenic potential activities in a wide diversity of in vitro and in vivo studies. The majority of the studies employed supraphysiological concentrations in vitro approaches, and numerous in vivo studies lacked detailed reporting of randomization, blinding, and extract characterization. The mechanistic evidence for saffron and its BACs on human cancer is largely based on gene or protein expression studies, which lack functional validation. Nevertheless, pharmacokinetic issues, poor bioavailability, and differences in the extracts’ composition, together with the lack of sufficient human randomized clinical trials, limit the clinical applicability of saffron and its BACs, although they appear to have potential biological acceptability.

### 4.3. Cardiovascular and Metabolic Disorders

Chronic hyperglycemia, oxidative stress, inflammation, endothelial dysfunction, mitochondrial damage, and insulin and vascular signaling pathway disregulations are just a few of the numerous pathomechanisms commonly seen in cardiovascular and metabolic diseases, such as diabetes mellitus, which remain an important global health concern with significant burdens. Type 2 diabetes mellitus contributes to the vast majority of prevalent cases due to of genetic predisposition as well as environmental factors [78]. Table 4 summarizes the relevant cardiometabolic protective effects of saffron and its main BACs.

Saffron-derived BACs modulate cholesterol metabolism at the level of lipid homeostasis through non-statin mechanisms, such as downregulation of PCSK9 and sortilin, suppression of SREBP-1/2 signaling, and upregulation of LDL receptor expression. In hypercholesterolemic models, these pathways were correlated with enhanced LDL clearance and declined hepatic inflammation without directly inhibiting 3-hydroxy-3-methylglutaryl (HMG)-CoA reductase [26,79].

Crocetin has demonstrated protective effects in diabetes-related vascular and renal complications. By reducing advanced glycation products, oxidative stress, and inflammatory mediators, suppressing transforming growth factor-b1 (TGF-β1) signaling, and restoring glyoxalase-I and paraoxonase-1 activities, crocetin decreased diabetic nephropathy and improved insulin resistance, dyslipidemia, and renal structural integrity [21].

Saffron supplementation, especially when combined with resistance training, improved lipid profiles and reduced pro-inflammatory adipokines such as leptin and resistin, supporting improved endothelial and cardiometabolic health in hypertensive individuals. These systemic metabolic benefits were also extended to vascular inflammation and cardiovascular risk modulation in humans [82]. Additionally, saffron improved wound healing by stimulating angiogenesis, fibroblast migration, collagen deposition, and VEGF overexpression in diabetic wound models, suggesting potential benefits in microvascular dysfunction [86].

The hypoglycemic and cardioprotective effects of saffron and its BACs were demonstrated through preclinical research, mainly via antioxidant, anti-inflammatory, and pathway-modulating activities. These studies were carried out on animal models (mainly STZ-induced models), which did not reflect human disease well and lacked reporting of the approaches applied, such as randomization, blinding, and dose translation. As highlighted in recent comprehensive analyses of plant-derived antioxidants [88,89], discrepancies frequently arise between preclinical efficacy and human therapeutic benefit due to differences in bioavailability, metabolism, dosing, and disease heterogeneity.

Similarly, clinical trials have shown improvements in metabolic and cardiovascular (e.g., ox-LDL and MCP-1) markers, although results vary and were often short-term and surrogate outcomes, such as reduction in HbA1c, reducing blood pressure, and preventing cardiovascular events. Variability in formulation, dosage, and study duration, as well as population characteristics, also limited the overall comparability of clinical trial results.

## 5. Pharmacokinetics, Bioavailability, Formulation Challenges, and Toxicological Considerations of Saffron BACs

Saffron and its BACs demonstrate a pharmacological fingerprint, but pharmacokinetic restrictions, related to absorption, bioavailability, and tissue distribution, restrict their practical translation.

Crocin is a hydrophilic, glycosylated metabolite with limited intestinal absorption in its natural state. The most absorbable and systemically accessible metabolite of crocin is its aglycone, crocetin, which is quickly hydrolyzed by intestinal epithelial enzymes and gut microbes after oral treatment [15,90]. As a result, crocetin is frequently recognized as the primary mediator of saffron’s systemic biological effects, being easily absorbed and measurable in plasma within 1–2 h after ingestion and being able to overcome biological barriers, according to in vivo pharmacokinetic studies.

Preclinical data support crocetin’s neuroprotective efficacy in conditions such as Alzheimer’s disease, Parkinson’s disease, cerebral ischemia, and multiple sclerosis partly due to its capacity to achieve central nervous system compartments either intrinsically or through formulation-assisted delivery [15,90,91]. However, fast metabolism, short and formulation-dependent plasma half-life, and interindividual variability in absorption remain a frontier in the global bioavailability of saffron BACs.

**Figure 2:**
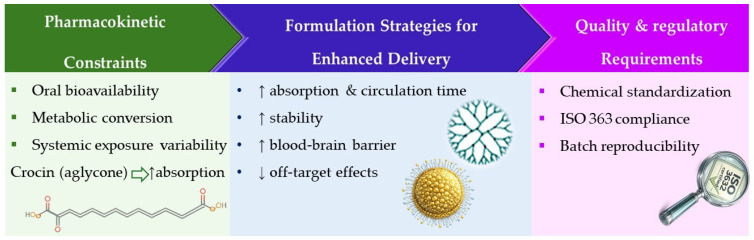
Translational pathway from pharmacokinetic constraints to formulation approaches, and regulatory standardization of saffron BACs.

Advanced formulation techniques have been investigated to overcome these matters. In preclinical models, nanoformulations, such as nanoparticles loaded with crocin or crocetin, nanoemulsions, liposomes, cyclodextrin inclusion complexes, and polymeric carriers, have shown improved chemical stability, cellular uptake, extended circulation time, and improved targeting [91,92,93]. In oncology and neurodegeneration models, nanoparticle-based delivery systems improved blood–brain barrier penetration and increased tumor cells’ sensitivity to conventional chemotherapeutic agents. This improves therapeutic efficacy while possibly lowering required doses and off-target effects. These results, however, remain mostly preclinical and require additional clinical validation (Figure 2).

From a clinical perspective, doses up to 200–400 mg/day of whole saffron extract have been used in cardiometabolic and inflammatory conditions, while human-relevant dosing ranges for saffron preparations usually fall between 15 and 60 mg/day for standardized extracts in neuropsychiatric and neurological trials [90,94]. Saffron demonstrates an appropriate safety and tolerability outline across these dosage ranges, with side effects similar to those of a placebo or traditional medications [90]. Crucially, when properly designed, formulation-enhanced delivery systems do not show increased cytotoxicity in neural or peripheral cell models, demonstrating their translational safety [91,92,94].

Variability in extract composition remains a main barrier to pharmaceutical development and regulatory approval. Cultivar, geographic origin, harvesting conditions, and post-harvest processing have a meaningful influence on the chemical composition of saffron. Therefore, to guarantee repeatable pharmacokinetics, effectiveness, and safety, quality control and standardization techniques are necessary. In addition to compliance with ISO saffron grading standards (ISO 3632), which categorize saffron quality based on spectrophotometric indices of coloring strength (crocin), aroma (safranal), and bitterness (picrocrocin), current regulatory frameworks place an emphasis on chemical standardization based on key marker compounds. Given its function as the metabolic precursor of crocetin and its direct impact on systemic exposure and central nervous system availability, crocin content is becoming more widely acknowledged as one of these markers [91,92,95]. Perhaps ISO 3632 was initially developed for culinary quality evaluation; hence, the establishment of *pharmacopoeial* standards specifically designed for medicinal extracts, considering the BAC’s concentration, purity, and limits of contaminants, as well as batch-to-batch consistency, will be crucial to support pharmaceutical development and clinical translation.

## 6. Conclusions

The pharmacological potential of saffron arises from its unique profile of BACs, namely crocin, crocetin, safranal, and picrocrocin. These BACs play a principal role in mediating antioxidant, anti-inflammatory, neuroprotective, and metabolic effects, while safranal contributes relevant neuromodulatory and cytoprotective properties.

According to preclinical and clinical evidence, these BACs may be beneficial for a wide range of non-communicable diseases. However, several challenges still limit the clinical use of BACs from saffron, including heterogeneity in results and weakened clinical conclusions. Future research should focus on standardizing uses of saffron extract preparation or its BACs in doses and improving formulation, also using long-term clinical trials to obtain reliable and more accurate results that can be compared and properly evaluated. Establishing rigorous research standards will be decisive in determining whether saffron can transition from a promising phytochemical source to a clinically actionable pharmacological entity.

## Figures and Tables

**Table 1:** Main BACs of saffron with pharmaceutical relevance, mechanisms of action, and therapeutic applications.

BACs	Main Mechanisms of Action	Pharmacological/Therapeutic Relevance	Refs.
**Crocins**	✓ Antioxidant activity via ROS scavenging; ✓ Modulation of NF-κB and Nrf2 signaling pathways; ✓ Anti-inflammatory effects through cytokine regulation; ✓ Inhibition of apoptosis and dysregulated cell proliferation	✓ Neuroprotection; ✓ Anticancer activity; ✓ Cardioprotection; ✓ Anti-inflammatory disorders and immune-mediated disorders	[7,9,10,12,13,18,19,20]
**Crocetin**	✓ Regulation of oxidative stress and inflammation response; ✓ Modulation of PI3K/Akt and MAPK signaling pathways; ✓ Improvement of mitochondrial function and bioenergetics; ✓ Enhanced tissue oxygen diffusion and microcirculation	✓ Supportive cancer therapy; ✓ Cardiovascular and cerebrovascular diseases; ✓ Neurodegenerative disorders; ✓ Ischemia-related conditions	[7,9,15,19,20,21,22]
**Safranal**	✓ Modulation of GABAergic and serotonergic neurotransmission; ✓ Antioxidant and anti-apoptotic effects; ✓ Regulation of neuronal excitability and synaptic signaling	✓ Depression and anxiety disorders; ✓ Epilepsy; ✓ Neurodegenerative diseases; ✓ Antioxidant-based neuroprotection	[7,9,19,23,24,25]
**Picrocrocin**	✓ Mild antioxidant and anti-inflammatory activity; ✓ Indirect contribution through bioconversion to safranal ✓ Potential synergistic interaction with other saffron constituents	✓ Supportive role in anti-inflammatory and antioxidant responses; ✓ Contribution to overall saffron bioactivity rather than standalone therapeutic use	[7,9,19,26,27]

Abbreviations: GABAergic, neurotransmitter gamma-aminobutyric acid; MAPK, mitogen-activated protein kinase; NF-κB, nuclear factor κB; Nrf2, nuclear factor erythroid 2-related factor 2; PI3K/Akt, phosphatidylinositol 3-kinase/protein kinase B; and ROS, reactive oxygen species.

**Table 2:** Neuroprotective and psychotropic effects of saffron BACs: evidence from in vitro, in vivo, and clinical studies.

Study Model	BAC/Extract	Dose (Duration)	Main Outcomes	Proposed Mechanisms	Ref.
**Alzheimer’s disease**					
Aβ_1–40_ aggregation model	trans-Crocin 4	320 µM	Altered Aβ monomer/ oligomer distribution	Redirection of Aβ aggregation towards non-toxic species	[33]
Microglial and neuron cells	Crocetin	25 µM (6–96 h)	↑ Aβ clearance	Autophagy induction via STK11/LKB1–AMPK signaling	[22]
AlCl_3_/ d-gal mice	Crocin	5–20 mg/kg/day (8 weeks); HDE (25–100 mg/day)	↓ Aβ deposition; ↑ antioxidant enzymes	Antioxidant and cholinergic modulation	[34]
Aβ_1–42_ rats	Crocin	30 mg/kg/day (12 days); HDE (290 mg/day)	↑ cognitive performance; ↓ neuronal apoptosis	Inhibition of c-Fos-dependent apoptotic signaling	[35]
C57BL/6J mice	Saffron formulation (Repron^®^)	10 mg/kg/day (20 days); HDE (50 mg/day)	↓ β-amyloid accumulation; partial modulation of neuroinflammation;	Anti-inflammatory and antioxidant activity; modulation of microglial activation	[8]
Clinical (n = 60; DBPC)	Saffron	15 mg twice daily (12 weeks)	↓ IL-1β and MDA; ↑ TAC	Systemic antioxidant and anti-inflammatory effects	[20]
**Parkinson’s disease**					
Pb-intoxicated rodents	Saffron extract	50 mg/kg/day (3 days); HDE (480 mg/day)	↑ dopaminergic integrity and locomotor function	Neuroprotection against heavy-metal-induced oxidative damage	[36]
Clinical (RCT, n = 53)	Crocin	60 mg/day (8 weeks)	↓ movement disorder severity; ↑ activities of daily living;	Dopaminergic modulation and antioxidant activity	[37]
Rotenone rats	Crocin	30 mg/kg/day (30 days); HDE (290 mg/day)	↑ motor function; ↓ α-synuclein accumulation	PI3K/Akt/mTOR modulation; miRNA regulation	[38]
**Multiple sclerosis**					
Cuprizone mice	Crocin	100 mg/kg, 3×/week (5 weeks); HDE (480 mg/day)	↑ motor coordination and behavioral performance	Antioxidant and anti-inflammatory effects	[39]
Clinical (RCT, n = 60)	Crocin-selenium nanoparticles	1 capsule/day (12 weeks)	↑ cognitive performance; ↑ TAC; no change in lipid peroxidation or systemic inflammation	Restoration of redox homeostasis; selenoprotein-mediated neuroprotection	[40]
Clinical (RCT, n = 43)	Saffron	500 mg/day (12 months)	↓ MMP-9; ↑ TIMP-1	Modulation of extracellular matrix remodeling and immune cell trafficking	[41]
**Cerebral ischemia**					
Ischemia/reperfusion rats	Saffron extract	100–200 mg/kg/day (3 weeks); HDE (960–1900 mg/day)	↓ apoptosis and lipid peroxidation	Antioxidant and proangiogenic modulation	[42]
Late phase ischemia rats	Saffron extract	30–300 mg/kg/day (40 days); HDE (290–2900 mg/day)	↑ neurological and cognitive outcomes	Anti-inflammatory and anti-gliotic effects	[43]
Clinical (RCT, n = 39)	Saffron extract	200 mg/day (4 days)	↓ stroke severity; ↑ BDNF	Neurotrophic and neuroprotective effects	[44]
**Mental illness**					
PC12 cells	Crocin	1–30 µM	↑ neuronal survival and morphology	Neurotrophic and antioxidant effects	[45]
Ex vivo human neurons	Saffron-derived metabolites	300 mg (oral dose)	Neuroprotection; ↑ BDNF	Monoaminergic modulation and neurotrophic signaling	[46]
Depression animal models	Saffron extract	50–200 mg/kg; HDE (480–1900 mg/day)	↓ depressive-like behaviors	Antianhedonic and antioxidant effects	[47]
Clinical (n = 48, DBPC)	Saffron extract	30 mg/day (6 weeks)	↑ perceived mental health; ↓ N-acetyl-phenylalanine; equal depressive/anxiety scores	Modulation of amino-acid metabolism and neuromodulator pathways	[48]
Clinical (RCT, n = 50)	Saffron	30–60 mg/day	↓ depressive symptoms	Serotonergic modulation	[49]

Abbreviation: AlCl_3_, aluminum chloride; Akt, protein kinase B; AMPK, AMP-activated protein kinase; Aβ, amyloid beta; BDNF, brain-derived neurotrophic factor; DBPC, double-blind placebo-controlled; IL-1β, interleukin-1β; MDA, malondialdehyde; MMP, matrix metalloproteinase; PI3K, phosphoinositide 3-kinase; RCT, randomized controlled trial; STK11, serine/threonine kinase 11; TAC, total antioxidant capacity; TIMP, tissue inhibitor of metalloproteinase-1. Human-equivalent doses (HED) were calculated using standard body surface area conversion factors when possible.

**Table 3:** Anticancer effects of saffron BACs: evidence from in vitro and in vivo studies.

Study Model	BAC/Extract	Dose (Duration)	Main Outcomes	Proposed Mechanisms	Refs.
**Breast cancer**					
MDA-MB-231 cells, BALB/c mice	Crocin; Crocetin	150 mg/kg/day (4 weeks); HDE (730 mg/day)	↓ lipid accumulation; ↓ tumor burden	Metabolic disruption; antiproliferative effects	[52]
MCF-7, 293T, MDA-MB-231 cells	Crocin	2.7–3 mM	Induction of apoptosis	ROS generation; FOXO3a nuclear translocation	[53]
MDA-MB-231 cells	Crocin + Doxorubicin	1.5–6 µM; 0.25–6 µM (24–48 h)	↑ doxorubicin cytotoxicity	Chemosensitization via apoptosis induction	[54]
MDA-MB-231, BT-549, MCF-7 cells	Crocin	0.5–4 mg/mL	Suppressed cell viability and proliferation	PRKCQ downregulation; NF-κB inhibition	[13]
MDA-MB-231, MDA-MB-468 cells	Safranal	30 min–24 h	Anti-proliferative and apoptotic effects	Mitochondrial dysfunction; metabolic inhibition	[55]
4T1 cells, BALB/c mice	Crocin + Crocetin	2–2.5 mM; 0.05–0.1 mM (3×/week)	↓ migration, invasion, and adhesion	Wnt/β-catenin signaling modulation; anti-metastatic synergy	[56]
4T1 cells, BALB/c mice	Crocin + Metformin	0–4.5 mM; 0–20 mM (3×/week)	↓ viability and migration; ↑ survival	Metabolic interference; VEGF and MMP-9 downregulation	[57]
**Prostate cancer**					
SiHa cells; BALC/c mice	Saffron extract	0–4 mg/mL	↓ proliferation; induced apoptosis	DNA methyltransferase downregulation	[29]
PCa cells, BALB/c mice	Safranal	100 mg/kg/day (24–32 h); HDE (490 mg/day)	Suppressed cell cycle re-entry	CDK2/4/6; Akt; NF-κB inhibition	[24]
**Cervical cancer**					
SiHa cells, BALB/c mice	Crocin	50 mg/kg/day (4 weeks); HDE (245 mg/day)	↓ viability and invasion; ↑ autophagy	AMPK/mTOR activation; antiproliferative effects	[58]
HeLa cells	Crocin	5–10 mM	Disruption of spindle microtubule dynamics	Mitotic disruption and apoptosis	[59]
**Skin cancer**					
BALB/c mice	Crocin	50 mg/kg/day (3 weeks); HDE (245 mg/day)	↓ tumor growth	Apoptosis induction	[60]
**Liver cancer**					
HepG2, HCC rats	Crocin + Sorafenib	50 + 50 mg/kg/day (6 weeks); HDE (490 mg/day)	↑ antitumor effects and hepatoprotection	Suppression of inflammation and oxidative stress	[61]
HCC rats	Crocin	10 mg/kg/day (4 weeks); HDE (1.6 mg/day)	↓ tumor progression and liver injury	Nrf2/HO-1 activation; Keap1 suppression	[62]
**Thyroid cancer**					
TPC-1, IHH-4 cells	Crocin	0–40 µM	↓ viability; apoptosis induction	miR-34a-5p/PTPN4 axis; ROS induction	[63]
FTC-133 cells	Crocin	0–45 µM	Anti-proliferative and apoptotic effects	ERK and STAT/JAK inhibition	[64]
8305c, TPC-1 cells	Crocin	0–40 µM	Suppressed proliferation; apoptosis	PI3K/Akt inhibition	[12]
**Colorectal cancer**					
HT-29, Caco-2 cells; NCR nu/nu mice	Crocin	0–40 µM	↓ proliferation, migration, invasion; inhibited angiogenesis	TNF-α/NF-κB/VEGF blockade; anti-angiogenic activity	[65]
CT26, HCT16 cells	Saffron extract	25–400 µg/mL	↓ tumor growth; ↓ proliferation; ↑ immunotherapy efficacy	↓ PD-1/PD-L1-mediated immune evasion; T-cell activation	[66]
Colo-205 cells	Safranal	0–200 µM	Cell cycle arrest; apoptosis	PI3K/Akt/mTOR inhibition	[67]
MMR-deficient HCT116 variants	Saffron; Safranal; Crocin	0–15 mg/mL; 0–900 µM; 0–1000 µM	Suppressed proliferation; apoptosis	CDC25b inhibition; caspase activation	[68]
SW480, SW620 cells	Saffron extracts	1–10%	↓ proliferation and migration	MACC1-dependent regulation	[69]
SW-480 cells	Crocin + Curcumin	1–50 µM	↑ chemosensitivity; ↓ inflammation	Oxidative stress modulation	[70]
**Gastric cancer**					
AGS cells	Saffron extract	20–100 µg/mL	↓ viability; ↑ apoptosis	Downregulation of stemness-associated genes	[71]
Balb/c nude mice	Crocin	6.25 mg/kg/day; HDE (30 mg/day)	↓ tumor progression	TPM4 modulation	[72]
EPG85-257RDB, EPG85-257 cells	Crocin + Doxorubicin	0–100 µM; 0–500 nM	↑ doxorubicin cytotoxicity	Chemosensitization via apoptosis induction	[73]
**Pancreatic cancer**					
Capan-2 cells, NCR nu/nu mice	Crocin	50–100 mg/kg; HDE (245–490 mg/day)	↓ viability; induced apoptosis	Caspase activation; mitochondrial dysfunction	[74]
**Brain cancer**					
U251, U373, U138; CD1-nu/nu mice	Crocetin	100 mg/kg (35 days); HDE (490 mg/day)	↓ tumor growth; ↑ survival	Antiproliferative effects	[75]
U87-MG cells	Crocin + CAPE	1–13 mM; 10–110 µM	↑ apoptosis	p53 activation; PCNA downregulation	[76]
**Lung cancer**					
A549, QU-DB cells	Saffron extract + Cisplatin	500–5000 µg/mL; 0–50 µM	↑ cisplatin cytotoxicity	ROS modulation; apoptosis induction	[77]

Abbreviation: Akt, protein kinase B; AMPK, AMP-activated protein kinase; CDC25b, cell division cycle 25B; CDK, cyclin-dependent kinase; ERK, extracellular signal-regulated kinase; FOXO3a, forkhead box O3a; JAK, Janus kinase; Keap1, Kelch-like ECH-associated protein 1; MACC1, metastasis-associated in colon cancer 1; MMP, matrix metalloproteinase; NF-κB, nuclear factor kappa B; Nrf2/HO-1, Nuclear factor erythroid 2-related factor/heme oxygenase-1; PCNA, proliferating cell nuclear antigen; PI3K, phosphoinositide 3-kinase; PRKCQ, protein kinase C theta; PTPN4, tyrosine-protein phosphatase non-receptor type 4; ROS, reactive oxygen species; STAT, signal transducer and activator of transcription; TPM4, tropomyosin-4; TNF-α, tumor necrosis factor; VEGF, vascular endothelial growth factor; Wnt, Wingless/integrated signaling pathway. Human-equivalent doses (HED) were calculated using standard body surface area conversion factors when possible.

**Table 4:** Pharmacological effects of saffron extracts and BACs in cardiovascular and metabolic disorders.

Study Model	BAC/Extract	Dose (Duration)	Main Outcomes	Proposed Mechanisms	Refs.
**Cardiovascular disorders**					
HepG2 cells	Picrocrocin	1–300 µg/mL (24–72 h)	↓ cholesterol synthesis; ↓ triglyceride synthesis; ↑ LDLR expression	Modulation of SREBP-2 activation; LDLR-mediated LDL uptake; regulation of HMGR, GPAT expression	[26]
C57BL/6 mice	Saffron extract	50 mg/kg/day (12 weeks); HDE (245 mg/day)	↓ cholesterol synthesis; ↓ triglyceride synthesis; ↑ LDLR expression	PCSK9 inhibition; upregulation of LDLR expression; modulation of SREBP-2 and SREBP-1c signaling	[79]
Hypertensive rat models	Saffron extract	10–40 mg/kg; HDE (100–390 mg/day)	↓ systolic and diastolic blood pressure	Inhibition of the renin–angiotensin system; vascular relaxation	[80]
Coronary artery disease patients	Crocin/ Saffron extract	30 mg/day (8 weeks)	↓ ox-LDL; ↑ SIRT1 and AMPK expression	Suppression of NF-κB signaling; improvement of metabolic and endothelial regulation	[81]
Hypertensive patients	Saffron extract	200 mg/day (12 weeks)	↓ inflammatory markers; improved lipid profile	Anti-inflammatory effects synergy with physical exercise	[82]
**Diabetes mellitus**					
STZ-induced diabetic rats	Saffron extract	25–100 mg/kg/day (21 days); HDE (245–970 mg/day)	↓ blood glucose; ↑ insulin levels; ↑ β-cell function	Modulation of glucose metabolism enzymes (↓ G6Pase, ↑ GK)	[83]
STZ-induced diabetic mice	Saffron extract	80 mg/kg/day (45 days); HDE (390 mg/day)	↓ blood glucose; ↓ caspase-3; ↑ renal histological	Anti-apoptotic and antioxidant effects	[84]
STZ-induced diabetic rats	Crocin; Sitagliptin	10 mg/kg/day (4 weeks); HDE (100 mg/day)	↑ glucose reduction and β-islet restoration	Antioxidant, anti-inflammatory, and anti-apoptotic actions	[85]
STZ-induced diabetic rats DN	Crocetin	100 mg/kg/day (3 months); HDE (970 mg/day)	↑ renal function; ↓ oxidative stress and inflammation	Downregulation of TGF-β1 signaling; antifibrotic effects	[21]
STZ-induced diabetic rats	Saffron extract	0–320 µg/mL (24–72 h)	↑ cell migration; ↑ angiogenesis; ↑ collagen deposition;	VEGF-dependent angiogenesis; suppression of TNF-α/NF-κB-mediated inflammation	[86]
T2DM patients	Saffron	100–400 mg/day (8–12 weeks)	↓ glycemia and dyslipidemia; ↑ quality of life	Anti-inflammatory and antioxidant effects	[87]

Abbreviation: AMPK, AMP-activated protein kinase; G6Pase, glucose 6-phosphatase; GK, glucokinase; GPAT, glycerol-3-phosphate acyltransferase; HMGR, 3-hydroxy-3-methylglutaryl-CoA reductase; LDLR, low-density lipoprotein receptor; NF-κB, nuclear factor kappa; ox-LDL, oxidized low-density lipoprotein-cholesterol; PCSK9, proprotein convertase subtilisin/kexin type 9; SIRT1, sirtuin 1; SREBP, sterol regulatory element-binding protein; STZ, streptozotocin; T2DM, type 2 diabetes mellitus; TGF-β1, transforming growth factor beta-1; TNF-α, tumor necrosis factor alpha; VEGF, vascular endothelial growth factor. Human-equivalent doses (HED) were calculated using standard body surface area conversion factors when possible.

## Data Availability

No new data were created or analyzed in this study. Data sharing is not applicable to this article.

## Abbreviations

The following abbreviations are used in this manuscript:

Aβ	amyloid beta
BACs	bioactive compounds
BDNF	brain-derived neurotrophic factor
G6Pase	glucose 6-phosphatase
GABAergic	gamma-aminobutyric acid neurotransmission-related
HMGR	3-hydroxy-3-methylglutaryl-CoA reductase
MACC1	metastasis-associated in colon cancer 1
MAPK	mitogen-activated protein kinase
MMP	matrix metalloproteinase
NF-κB	nuclear factor kappa B
Nrf2	nuclear factor erythroid 2-related factor 2
PCNA	proliferating cell nuclear antigen
PCSK9	proprotein convertase subtilisin/kexin type 9
PI3K/Akt	phosphatidylinositol 3-kinase/protein kinase B
SIRT1	sirtuin 1
SSRI	selective serotonin reuptake inhibitors
TGF-β1	transforming growth factor beta-1
TIMP	tissue inhibitor of metalloproteinase-1
TNF-α	tumor necrosis factor alpha
VEGF	vascular endothelial growth factor
